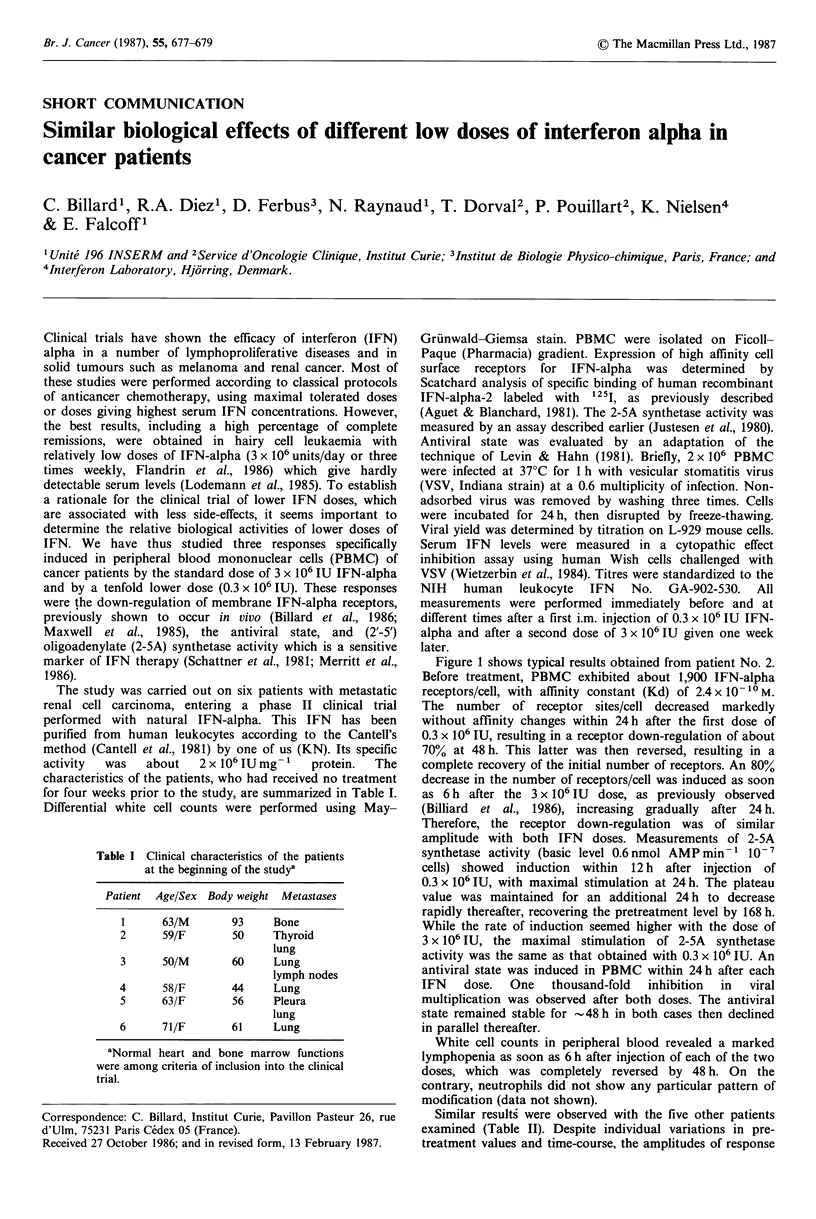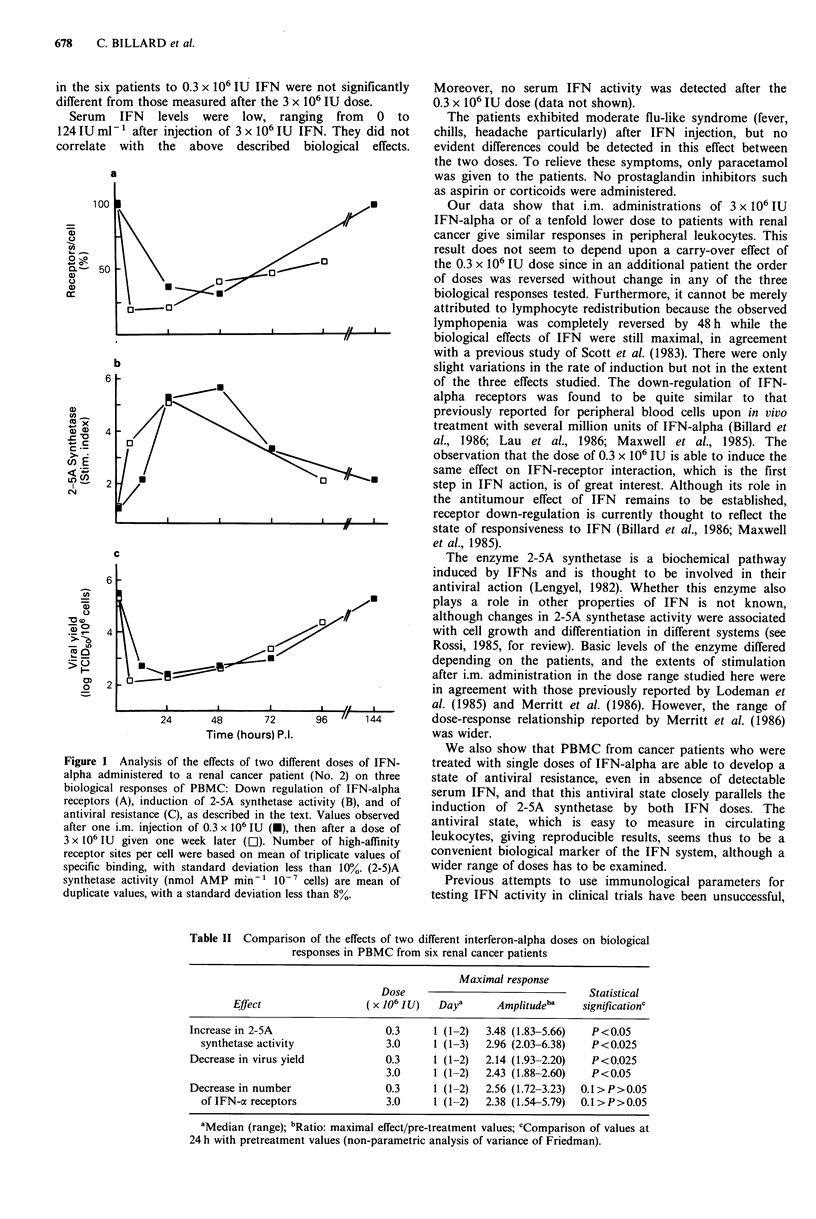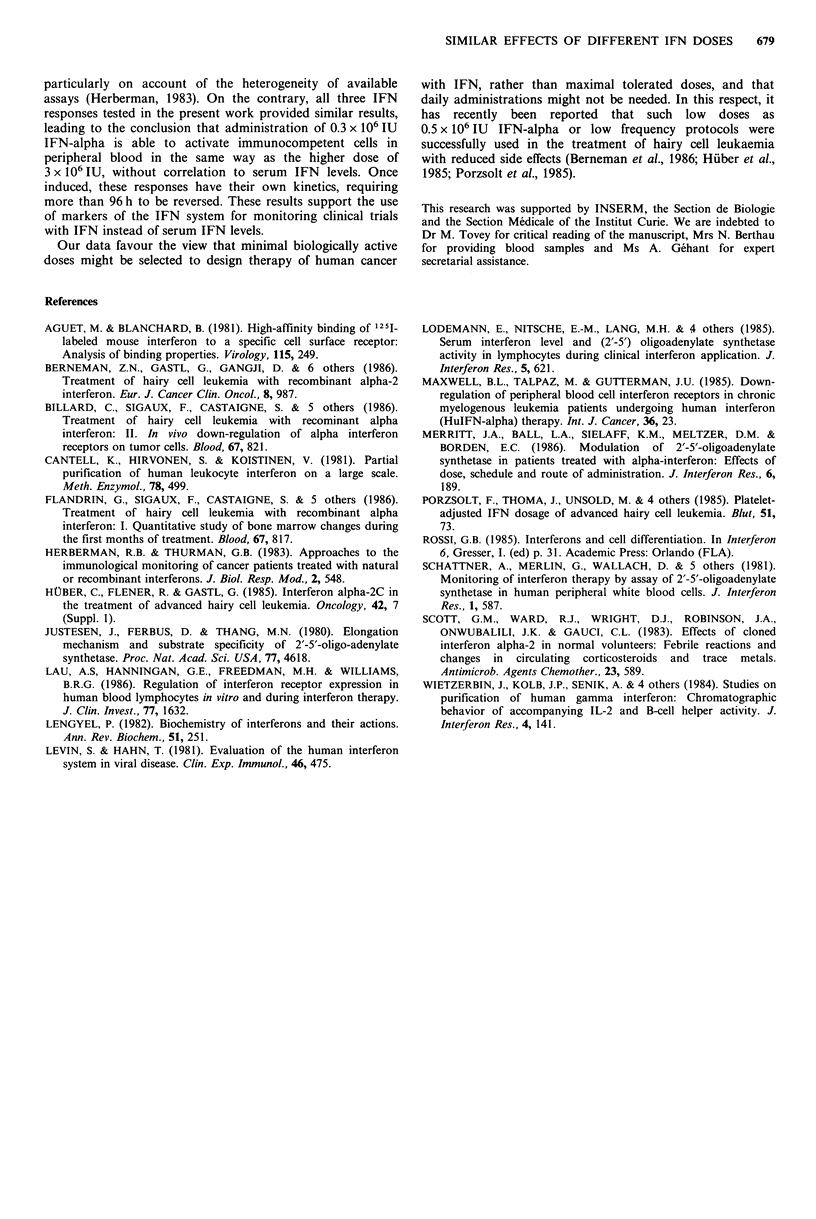# Similar biological effects of different low doses of interferon alpha in cancer patients.

**DOI:** 10.1038/bjc.1987.138

**Published:** 1987-06

**Authors:** C. Billard, R. A. Diez, D. Ferbus, N. Raynaud, T. Dorval, P. Pouillart, K. Nielsen, E. Falcoff


Br. J. Cancer (1987), 55, 677–679 © The Macmillan Press Ltd., 1987

SHORT COMMUNICATION

### Similar biological effects of different low doses of interferon alpha in cancer patients

C. Billard[1], R.A. Diez[1], D. Ferbus[3], N. Raynaud[1], T. Dorval[2], P. Pouillart[2], K. Nielsen[4] & E. Falcoff[1]

[1]*Unité 196 INSERM and* [2]*Service d'Oncologie Clinique, Institut Curie;* [3]*Institut de Biologie Physico-chimique, Paris, France; and* [4]*Interferon Laboratory, Hjörring, Denmark.*

Clinical trials have shown the efficacy of interferon (IFN) alpha in a number of lymphoproliferative diseases and in solid tumours such as melanoma and renal cancer. Most of these studies were performed according to classical protocols of anticancer chemotherapy, using maximal tolerated doses or doses giving highest serum IFN concentrations. However, the best results, including a high percentage of complete remissions, were obtained in hairy cell leukaemia with relatively low doses of IFN-alpha ($3 \times 10^6$ units/day or three times weekly, Flandrin *et al.*, 1986) which give hardly detectable serum levels (Lodemann *et al.*, 1985). To establish a rationale for the clinical trial of lower IFN doses, which are associated with less side-effects, it seems important to determine the relative biological activities of lower doses of IFN. We have thus studied three responses specifically induced in peripheral blood mononuclear cells (PBMC) of cancer patients by the standard dose of $3 \times 10^6$ IU IFN-alpha and by a tenfold lower dose ($0.3 \times 10^6$ IU). These responses were the down-regulation of membrane IFN-alpha receptors, previously shown to occur *in vivo* (Billard *et al.*, 1986; Maxwell *et al.*, 1985), the antiviral state, and (2′-5′) oligoadenylate (2-5A) synthetase activity which is a sensitive marker of IFN therapy (Schattner *et al.*, 1981; Merritt *et al.*, 1986).

The study was carried out on six patients with metastatic renal cell carcinoma, entering a phase II clinical trial performed with natural IFN-alpha. This IFN has been purified from human leukocytes according to the Cantell's method (Cantell *et al.*, 1981) by one of us (KN). Its specific activity was about $2 \times 10^6\,IU\,mg^{-1}$ protein. The characteristics of the patients, who had received no treatment for four weeks prior to the study, are summarized in Table I. Differential white cell counts were performed using May–Grünwald–Giemsa stain. PBMC were isolated on Ficoll–Paque (Pharmacia) gradient. Expression of high affinity cell surface receptors for IFN-alpha was determined by Scatchard analysis of specific binding of human recombinant IFN-alpha-2 labeled with $^{125}I$, as previously described (Aguet & Blanchard, 1981). The 2-5A synthetase activity was measured by an assay described earlier (Justesen *et al.*, 1980). Antiviral state was evaluated by an adaptation of the technique of Levin & Hahn (1981). Briefly, $2 \times 10^6$ PBMC were infected at 37°C for 1 h with vesicular stomatitis virus (VSV, Indiana strain) at a 0.6 multiplicity of infection. Non-adsorbed virus was removed by washing three times. Cells were incubated for 24 h, then disrupted by freeze-thawing. Viral yield was determined by titration on L-929 mouse cells. Serum IFN levels were measured in a cytopathic effect inhibition assay using human Wish cells challenged with VSV (Wietzerbin *et al.*, 1984). Titres were standardized to the NIH human leukocyte IFN No. GA-902-530. All measurements were performed immediately before and at different times after a first i.m. injection of $0.3 \times 10^6$ IU IFN-alpha and after a second dose of $3 \times 10^6$ IU given one week later.

**Table I** Clinical characteristics of the patients at the beginning of the study[a]

| *Patient* | *Age/Sex* | *Body weight* | *Metastases* |
|---|---|---|---|
| 1 | 63/M | 93 | Bone |
| 2 | 59/F | 50 | Thyroid lung |
| 3 | 50/M | 60 | Lung lymph nodes |
| 4 | 58/F | 44 | Lung |
| 5 | 63/F | 56 | Pleura lung |
| 6 | 71/F | 61 | Lung |

[a]Normal heart and bone marrow functions were among criteria of inclusion into the clinical trial.

Figure 1 shows typical results obtained from patient No. 2. Before treatment, PBMC exhibited about 1,900 IFN-alpha receptors/cell, with affinity constant (Kd) of $2.4 \times 10^{-10}$ M. The number of receptor sites/cell decreased markedly without affinity changes within 24 h after the first dose of $0.3 \times 10^6$ IU, resulting in a receptor down-regulation of about 70% at 48 h. This latter was then reversed, resulting in a complete recovery of the initial number of receptors. An 80% decrease in the number of receptors/cell was induced as soon as 6 h after the $3 \times 10^6$ IU dose, as previously observed (Billiard *et al.*, 1986), increasing gradually after 24 h. Therefore, the receptor down-regulation was of similar amplitude with both IFN doses. Measurements of 2-5A synthetase activity (basic level 0.6 nmol AMP $min^{-1}$ $10^{-7}$ cells) showed induction within 12 h after injection of $0.3 \times 10^6$ IU, with maximal stimulation at 24 h. The plateau value was maintained for an additional 24 h to decrease rapidly thereafter, recovering the pretreatment level by 168 h. While the rate of induction seemed higher with the dose of $3 \times 10^6$ IU, the maximal stimulation of 2-5A synthetase activity was the same as that obtained with $0.3 \times 10^6$ IU. An antiviral state was induced in PBMC within 24 h after each IFN dose. One thousand-fold inhibition in viral multiplication was observed after both doses. The antiviral state remained stable for ~48 h in both cases then declined in parallel thereafter.

White cell counts in peripheral blood revealed a marked lymphopenia as soon as 6 h after injection of each of the two doses, which was completely reversed by 48 h. On the contrary, neutrophils did not show any particular pattern of modification (data not shown).

Similar results were observed with the five other patients examined (Table II). Despite individual variations in pre-treatment values and time-course, the amplitudes of response

Correspondence: C. Billard, Institut Curie, Pavillon Pasteur 26, rue d'Ulm, 75231 Paris Cédex 05 (France).
Received 27 October 1986; and in revised form, 13 February 1987.

in the six patients to $0.3 \times 10^6$ IU IFN were not significantly different from those measured after the $3 \times 10^6$ IU dose.

Serum IFN levels were low, ranging from 0 to 124 IU $ml^{-1}$ after injection of $3 \times 10^6$ IU IFN. They did not correlate with the above described biological effects.

**Figure 1** Analysis of the effects of two different doses of IFN-alpha administered to a renal cancer patient (No. 2) on three biological responses of PBMC: Down regulation of IFN-alpha receptors (A), induction of 2-5A synthetase activity (B), and of antiviral resistance (C), as described in the text. Values observed after one i.m. injection of $0.3 \times 10^6$ IU (■), then after a dose of $3 \times 10^6$ IU given one week later (□). Number of high-affinity receptor sites per cell were based on mean of triplicate values of specific binding, with standard deviation less than 10%. (2-5)A synthetase activity (nmol AMP $min^{-1}$ $10^{-7}$ cells) are mean of duplicate values, with a standard deviation less than 8%.

Moreover, no serum IFN activity was detected after the $0.3 \times 10^6$ IU dose (data not shown).

The patients exhibited moderate flu-like syndrome (fever, chills, headache particularly) after IFN injection, but no evident differences could be detected in this effect between the two doses. To relieve these symptoms, only paracetamol was given to the patients. No prostaglandin inhibitors such as aspirin or corticoids were administered.

Our data show that i.m. administrations of $3 \times 10^6$ IU IFN-alpha or of a tenfold lower dose to patients with renal cancer give similar responses in peripheral leukocytes. This result does not seem to depend upon a carry-over effect of the $0.3 \times 10^6$ IU dose since in an additional patient the order of doses was reversed without change in any of the three biological responses tested. Furthermore, it cannot be merely attributed to lymphocyte redistribution because the observed lymphopenia was completely reversed by 48 h while the biological effects of IFN were still maximal, in agreement with a previous study of Scott *et al.* (1983). There were only slight variations in the rate of induction but not in the extent of the three effects studied. The down-regulation of IFN-alpha receptors was found to be quite similar to that previously reported for peripheral blood cells upon *in vivo* treatment with several million units of IFN-alpha (Billard *et al.*, 1986; Lau *et al.*, 1986; Maxwell *et al.*, 1985). The observation that the dose of $0.3 \times 10^6$ IU is able to induce the same effect on IFN-receptor interaction, which is the first step in IFN action, is of great interest. Although its role in the antitumour effect of IFN remains to be established, receptor down-regulation is currently thought to reflect the state of responsiveness to IFN (Billard *et al.*, 1986; Maxwell *et al.*, 1985).

The enzyme 2-5A synthetase is a biochemical pathway induced by IFNs and is thought to be involved in their antiviral action (Lengyel, 1982). Whether this enzyme also plays a role in other properties of IFN is not known, although changes in 2-5A synthetase activity were associated with cell growth and differentiation in different systems (see Rossi, 1985, for review). Basic levels of the enzyme differed depending on the patients, and the extents of stimulation after i.m. administration in the dose range studied here were in agreement with those previously reported by Lodeman *et al.* (1985) and Merritt *et al.* (1986). However, the range of dose-response relationship reported by Merritt *et al.* (1986) was wider.

We also show that PBMC from cancer patients who were treated with single doses of IFN-alpha are able to develop a state of antiviral resistance, even in absence of detectable serum IFN, and that this antiviral state closely parallels the induction of 2-5A synthetase by both IFN doses. The antiviral state, which is easy to measure in circulating leukocytes, giving reproducible results, seems thus to be a convenient biological marker of the IFN system, although a wider range of doses has to be examined.

Previous attempts to use immunological parameters for testing IFN activity in clinical trials have been unsuccessful,

**Table II** Comparison of the effects of two different interferon-alpha doses on biological responses in PBMC from six renal cancer patients

| *Effect* | *Dose* ($\times 10^6$ *IU*) | *Maximal response* | | *Statistical* *signification*[c] |
|---|---|---|---|---|
| | | *Day*[a] | *Amplitude*[ba] | |
| Increase in 2-5A | 0.3 | 1 (1–2) | 3.48 (1.83–5.66) | $P<0.05$ |
| synthetase activity | 3.0 | 1 (1–3) | 2.96 (2.03–6.38) | $P<0.025$ |
| Decrease in virus yield | 0.3 | 1 (1–2) | 2.14 (1.93–2.20) | $P<0.025$ |
| | 3.0 | 1 (1–2) | 2.43 (1.88–2.60) | $P<0.05$ |
| Decrease in number | 0.3 | 1 (1–2) | 2.56 (1.72–3.23) | $0.1>P>0.05$ |
| of IFN-α receptors | 3.0 | 1 (1–2) | 2.38 (1.54–5.79) | $0.1>P>0.05$ |

[a]Median (range); [b]Ratio: maximal effect/pre-treatment values; [c]Comparison of values at 24 h with pretreatment values (non-parametric analysis of variance of Friedman).

particularly on account of the heterogeneity of available assays (Herberman, 1983). On the contrary, all three IFN responses tested in the present work provided similar results, leading to the conclusion that administration of $0.3 \times 10^6$ IU IFN-alpha is able to activate immunocompetent cells in peripheral blood in the same way as the higher dose of $3 \times 10^6$ IU, without correlation to serum IFN levels. Once induced, these responses have their own kinetics, requiring more than 96 h to be reversed. These results support the use of markers of the IFN system for monitoring clinical trials with IFN instead of serum IFN levels.

Our data favour the view that minimal biologically active doses might be selected to design therapy of human cancer with IFN, rather than maximal tolerated doses, and that daily administrations might not be needed. In this respect, it has recently been reported that such low doses as $0.5 \times 10^6$ IU IFN-alpha or low frequency protocols were successfully used in the treatment of hairy cell leukaemia with reduced side effects (Berneman *et al.*, 1986; Hüber *et al.*, 1985; Porzsolt *et al.*, 1985).

This research was supported by INSERM, the Section de Biologie and the Section Médicale of the Institut Curie. We are indebted to Dr M. Tovey for critical reading of the manuscript, Mrs N. Berthau for providing blood samples and Ms A. Géhant for expert secretarial assistance.

### References

AGUET, M. & BLANCHARD, B. (1981). High-affinity binding of $^{125}$I-labeled mouse interferon to a specific cell surface receptor: Analysis of binding properties. *Virology*, **115**, 249.

BERNEMAN, Z.N., GASTL, G., GANGJI, D. & 6 others (1986). Treatment of hairy cell leukemia with recombinant alpha-2 interferon. *Eur. J. Cancer Clin. Oncol.*, **8**, 987.

BILLARD, C., SIGAUX, F., CASTAIGNE, S. & 5 others (1986). Treatment of hairy cell leukemia with recominant alpha interferon: II. *In vivo* down-regulation of alpha interferon receptors on tumor cells. *Blood*, **67**, 821.

CANTELL, K., HIRVONEN, S. & KOISTINEN, V. (1981). Partial purification of human leukocyte interferon on a large scale. *Meth. Enzymol.*, **78**, 499.

FLANDRIN, G., SIGAUX, F., CASTAIGNE, S. & 5 others (1986). Treatment of hairy cell leukemia with recombinant alpha interferon: I. Quantitative study of bone marrow changes during the first months of treatment. *Blood*, **67**, 817.

HERBERMAN, R.B. & THURMAN, G.B. (1983). Approaches to the immunological monitoring of cancer patients treated with natural or recombinant interferons. *J. Biol. Resp. Mod.*, **2**, 548.

HÜBER, C., FLENER, R. & GASTL, G. (1985). Interferon alpha-2C in the treatment of advanced hairy cell leukemia. *Oncology*, **42**, 7 (Suppl. 1).

JUSTESEN, J., FERBUS, D. & THANG, M.N. (1980). Elongation mechanism and substrate specificity of 2′-5′-oligo-adenylate synthetase. *Proc. Nat. Acad. Sci. USA*, **77**, 4618.

LAU, A.S, HANNINGAN, G.E., FREEDMAN, M.H. & WILLIAMS, B.R.G. (1986). Regulation of interferon receptor expression in human blood lymphocytes *in vitro* and during interferon therapy. *J. Clin. Invest.*, **77**, 1632.

LENGYEL, P. (1982). Biochemistry of interferons and their actions. *Ann. Rev. Biochem.*, **51**, 251.

LEVIN, S. & HAHN, T. (1981). Evaluation of the human interferon system in viral disease. *Clin. Exp. Immunol.*, **46**, 475.

LODEMANN, E., NITSCHE, E.-M., LANG, M.H. & 4 others (1985). Serum interferon level and (2′-5′) oligoadenylate synthetase activity in lymphocytes during clinical interferon application. *J. Interferon Res.*, **5**, 621.

MAXWELL, B.L., TALPAZ, M. & GUTTERMAN, J.U. (1985). Down-regulation of peripheral blood cell interferon receptors in chronic myelogenous leukemia patients undergoing human interferon (HuIFN-alpha) therapy. *Int. J. Cancer*, **36**, 23.

MERRITT, J.A., BALL, L.A., SIELAFF, K.M., MELTZER, D.M. & BORDEN, E.C. (1986). Modulation of 2′-5′-oligoadenylate synthetase in patients treated with alpha-interferon: Effects of dose, schedule and route of administration. *J. Interferon Res.*, **6**, 189.

PORZSOLT, F., THOMA, J., UNSOLD, M. & 4 others (1985). Platelet-adjusted IFN dosage of advanced hairy cell leukemia. *Blut*, **51**, 73.

ROSSI, G.B. (1985). Interferons and cell differentiation. In *Interferon 6*, Gresser, I. (ed) p. 31. Academic Press: Orlando (FLA).

SCHATTNER, A., MERLIN, G., WALLACH, D. & 5 others (1981). Monitoring of interferon therapy by assay of 2′-5′-oligoadenylate synthetase in human peripheral white blood cells. *J. Interferon Res.*, **1**, 587.

SCOTT, G.M., WARD, R.J., WRIGHT, D.J., ROBINSON, J.A., ONWUBALILI, J.K. & GAUCI, C.L. (1983). Effects of cloned interferon alpha-2 in normal volunteers: Febrile reactions and changes in circulating corticosteroids and trace metals. *Antimicrob. Agents Chemother.*, **23**, 589.

WIETZERBIN, J., KOLB, J.P., SENIK, A. & 4 others (1984). Studies on purification of human gamma interferon: Chromatographic behavior of accompanying IL-2 and B-cell helper activity. *J. Interferon Res.*, **4**, 141.